# Breast Cancer Histopathology Image Classification Using an Ensemble of Deep Learning Models

**DOI:** 10.3390/s20164373

**Published:** 2020-08-05

**Authors:** Zabit Hameed, Sofia Zahia, Begonya Garcia-Zapirain, José Javier Aguirre, Ana María Vanegas

**Affiliations:** 1 eVida Research Group, University of Deusto, 48007 Bilbao, Spain; sofia.zahia95@deusto.es (S.Z.); mbgarciazapi@deusto.es (B.G.-Z.); 2 Biokeralty Reseach Institute, 01510 Vitoria, Spain; josej.aguirre@keralty.com; 3 Department of Pathological Anatomy, University Hospital of Araba, 01009 Vitoria, Spain; 4 Clinica Colsanitas, Bogotá 110221, Colombia; anmvanegas@colsanitas.com

**Keywords:** deep learning, histopathology, breast cancer, image classification, ensemble models

## Abstract

Breast cancer is one of the major public health issues and is considered a leading cause of cancer-related deaths among women worldwide. Its early diagnosis can effectively help in increasing the chances of survival rate. To this end, biopsy is usually followed as a gold standard approach in which tissues are collected for microscopic analysis. However, the histopathological analysis of breast cancer is non-trivial, labor-intensive, and may lead to a high degree of disagreement among pathologists. Therefore, an automatic diagnostic system could assist pathologists to improve the effectiveness of diagnostic processes. This paper presents an ensemble deep learning approach for the definite classification of non-carcinoma and carcinoma breast cancer histopathology images using our collected dataset. We trained four different models based on pre-trained VGG16 and VGG19 architectures. Initially, we followed 5-fold cross-validation operations on all the individual models, namely, fully-trained VGG16, fine-tuned VGG16, fully-trained VGG19, and fine-tuned VGG19 models. Then, we followed an ensemble strategy by taking the average of predicted probabilities and found that the ensemble of fine-tuned VGG16 and fine-tuned VGG19 performed competitive classification performance, especially on the carcinoma class. The ensemble of fine-tuned VGG16 and VGG19 models offered sensitivity of 97.73% for carcinoma class and overall accuracy of 95.29%. Also, it offered an F1 score of 95.29%. These experimental results demonstrated that our proposed deep learning approach is effective for the automatic classification of complex-natured histopathology images of breast cancer, more specifically for carcinoma images.

## 1. Introduction

Cancer is one of the critical public health issues around the world. According to the Global Burden of Disease (GBD) study, there have been 24.5 million cancer incidence and 9.6 million cancer deaths worldwide in 2017 [1]. These statistics indicate that cancer incidence expanded by 33% between 2007 and 2017 worldwide [1]. Specifically, breast cancer is the most common malignancy and the leading cause of cancer-related mortalities among women worldwide [1,2]. Thus, premature diagnosis of this pathology is crucial to preclude its progression and reduce its morbidity rates in women.

**Figure 1:**
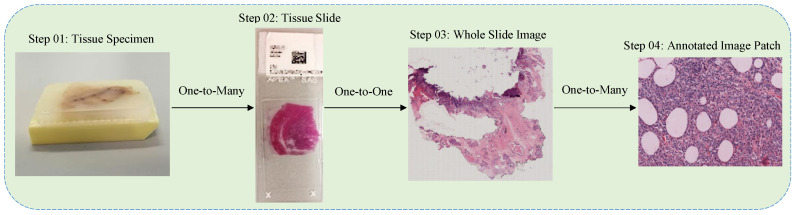
The complete process of biopsy is depicted in Figure. Steps 01 and 02 are taken from [7] whereas steps 03 and 04 are retrieved from our own dataset.

Breast cancer is a heterogeneous disease, composed of numerous entities with distinctive biological, histological and clinical characteristics [3]. This malignancy erupts from the growth of abnormal breast cells and might invade the adjacent healthy tissues [3]. Its clinical screening is initially performed by utilizing radiology images, for instance, mammography, ultrasound imaging, and Magnetic Resonance Imaging (MRI) [4,5]. However, these non-invasive imaging approaches may not be capable of determining the cancerous areas efficiently. To this end, the biopsy technique is usually used to analyze the malignancy in breast cancer tissues more comprehensively. The process of biopsy includes the collection of tissue samples, mounting them on microscopic glass slides, and staining these slides for better visualization of nuclei and cytoplasm [6]. Pathologists then carry out the microscopic analysis of these slides in order to finalize the diagnosis of breast cancer [6]. The complete process of biopsy technique is depicted in Figure 1, and is comprehensively described in [7].

However, the manual analysis of complex-natured histopathological images is fairly a time-consuming and tedious process, and could be prone to errors. Also, the morphological criteria used in the classification of these images are somehow subjective, which leads to the result that an average diagnostic concordance among the pathologists is approximately 75% [8]. Therefore, the computer-assisted diagnosis [4,6,9] plays a significant role to assist pathologists in analyzing the histopathology images. Specifically, it improves the diagnostic accuracy of breast cancer by reducing the inter-pathologist variations in diagnostic decisions [6]. However, the conventional computerized diagnostic approaches, ranging from rule-based systems to machine learning techniques, may not effectively challenge the intra-class variation and inter-class consistency within the histopathology images of breast cancer [10]. Also, these methodologies mainly rely on feature extraction methods like scale-invariant feature transform [11], speed robust features [12] and local binary patterns [13] which all are based on supervised information and can be prone to biased results during the classification of breast cancer histopathology images [10]. Therefore, the need for efficient diagnosis leads to an advanced set of computational models based on multiple layers of nonlinear processing units, called deep learning [14].

Recently, deep learning models [7,14,15,16,17] have made remarkable progress in computer vision, specifically in biomedical image processing, due to their abilities to automatically learn complicated and advanced features from images, which inspired various researchers to leverage these models in the classification of breast cancer histopathology images [7]. Especially convolutional neural networks (CNNs) [18] are widely used in image-related tasks due to their abilities to effectively share parameters across various layers within a deep learning model. Numerous CNN-based architectures have been proposed during the past few years; however, AlexNet [19] is considered as of the first deep CNNs to achieve considerable accuracy on the ImageNet Large Scale Visual Recognition Challenge (ILSVRC) during 2012. Thereafter, VGG architecture [20] introduced the idea of leveraging deeper networks with smaller convolutional filters, and achieved second place at ILSVRC 2014. The intuition of multiple stacked smaller convolutional filters can provide an effective receptive field and is also used in recently proposed pretrained models, including Inception Network [21] and residual neural network (ResNet) [22]. In this work, we employed two different approaches of VGG architecture for an efficient classification of breast cancer histopathology by utilizing our own created dataset. The main contributions of this paper are: First, we created a private dataset of whole slide images (WSI) from breast cancer patients with the help of experienced pathologists. Then, image patches were extracted from the WSI images, composed of non-carcinoma and carcinoma classes. Next, we selected and trained different combinations of pretrained VGG16 and VGG19 [20] deep learning architectures (discussed in Section 3). Specifically, we evaluated an individual as well as ensemble performances of fully-trained and fine-tuned VGG16 and VGG19 frameworks [20]. Of note, our main objective is the correct classification of the carcinoma class on a priority basis and we found that the ensemble of fine-tuned VGG16 and VGG19 approach [20] provided superior performance in the classification of non-carcinoma and carcinoma histopathology images of breast cancer.

The remaining sections of this paper are provided as follows. Section 2 presents related work. Section 3 demonstrates the materials and methods used to conduct this research. Section 4 shows experimental setup and Section 5 illustrates the results along with discussion. Finally, Section 6 highlights the conclusion and future direction of this study.

## 2. Related Work

With the evolution of machine learning in biomedical engineering, numerous studies leveraged handcrafted features-based approaches for the classification of histopathology images related to breast cancer. For instance, Kowal et al. [23] focused on the nuclei segmentation and extracted forty-two morphological, topological and texture features from the segmented nuclei of 500 fine-needle biopsy images of breast cancer. Then, these features were utilized to train three different classifiers in order to classify these images into benign and malignant classes. Similarly, Filipczuk et al. [24] also showed interest in the segmentation of nuclei and extracted twenty-five shape-based and texture-based features from the segmented nuclei of 737 cytology images of breast cancer. Based on these features, four different machine learning classifiers, namely, KNN (K-nearest neighbor), NB (Naive Bayes), DT (decision tree), and SVM (support vector machine), were trained for the classification of these cytological images into benign and malignant cases. Apart from nuclei segmentation [23,24], other studies focused on the extraction of global features from the whole images. For instance, Zhang et al. [25] combined local binary patterns, statistics from the gray level co-occurrence matrix and the curvelet transform, and designed a cascade random space ensemble scheme (with rejection options) for an efficient classification of the microscopic biopsy images of breast cancer. Although the traditional machine learning approaches have made satisfactory performances in analyzing the histological images of breast cancer, their performances mainly rely on the selection of features on which they are trained. Furthermore, they might not be capable of effectively extracting and organizing the discriminative information from data [26].

In contrast to the traditional machine learning approaches based on hand-crafted features, deep learning models have the ability to yield complicated and high-level features from images automatically [26]. Consequently, numerous recent studies employed deep learning approaches, with and without leveraging the pre-trained models, for the classification of breast cancer histopathology images. Of note, most of these studies employed BreakHis dataset [27] for the classification task. For instance, Spanhol et al. [28] employed CNN for the classification of breast cancer histopathology images and achieved 4 to 6 percentage points higher accuracy on BreakHis dataset [27] when using a variation of AlexNet [19]. Similarly, Bayramoglu et al. [29] utilized CNN in order to classify the histopathology images breast cancer irrespectively of their resolution using BreakHis dataset [27]. Specifically, the authors proposed single-task and multi-task CNN architectures; whereas the former was capable of predicting malignancy only and the latter was able to predict malignancy and magnification intensity of images simultaneously. These studies leveraging BreakHis dataset provided various state-of-the-art performances; however, they are relying on the same dataset. In this study, we followed the recent approaches of Araújo et al. [30] and Yan et al. [31] and presented a dataset for the classification of breast cancer histology images using deep learning models. However, our dataset contains only non-carcinoma and carcinoma classes, unlike [30,31] which have four classes in their classification problem. The explanation of our dataset and proposed methodologies are comprehensively discussed in the next Section 3.

## 3. Materials and Methods

In this section, we introduced our dataset, followed by its preprocessing methodology and training, validation, and testing criteria along with the augmentation process. Then, we discussed the layout of the VGG model and finally, we described the architecture of our proposed ensemble architecture.

### 3.1. Data Collection

We collected overall 544 whole slides images (WSI) from 80 patients suffering from breast cancer in the pathology department of Colsanitas Colombia University, Bogotá, Colombia. The tumor tissue fragments were fixed in formalin and embedded in paraffin. Subsequently, 4 mm cuts were made that were stained with hematoxylin and eosin (H & E). For the Immunohistochemistry studies, the paraffin-embedded tissue sections were treated with xylene to render them diaphanous (the paraffin being removed later by passing it through decreasing alcohol concentrations until 100% water was reached). Rehydrated sections were rinsed in phosphate buffered saline (PBS) containing 1% Tween-20. For the detection of proteins, sections were heated in a high pH Envision FLEX target retrieval solution at ${65\;}^{{}^{\circ}}C$ for 20 min and then incubated for 20 min at room temperature in the same solution. Endogenous peroxidase activity (3% $H_{2}$$O_{2}$) and non-specific binding (33% foetal calf serum) were blocked and the sections were incubated overnight at ${4\;}^{{}^{\circ}}C$ with the following primary antibodies: anti-ER (estrogen receptor), anti-PR (progesterone receptor), anti-HER-2 (human epidermal growth factor receptor-2), anti-myosin, anti-Ki-67 (proliferation-associated biomarker). Next, an Ultra View universal DAB kit was used following the manufacturer’s recommendations in conjunction with an automated staining procedure.

The tissue sections were then scanned at high resolution (400×) using a Roche iScan HT scanner (https://diagnostics.roche.com/global/en/products/instruments/ventana-iscan-ht.html). These WSI images representing multiple cases from every patient were analyzed using H & E, hormone receptors, including ER, PR, HER2, myosin, and Ki-67. Next, two pathologists examined the digital whole slides of tissue stained with H & E and extracted 845 areas from WSI, among which 408 are non-carcinoma and 437 are carcinoma images.The carcinoma class has images of malignant tumors whereas the non-carcinoma class contains images of normal tisues as well as benign images of non-tumor glandular tissues. These areas were photographed at 200× (50 micrometers of resolution) and exported to png format using Qupath 0.1.2 software [32]. The dimensions of these images were noted as 1278×760 pixels. This dataset is considered to be balanced and its statistics are represented in Table 1. The main objective related to this dataset is the automatic classification of breast cancer histopathology images, most importantly the carcinoma images.

### 3.2. Preprocessing

**Figure 2:**
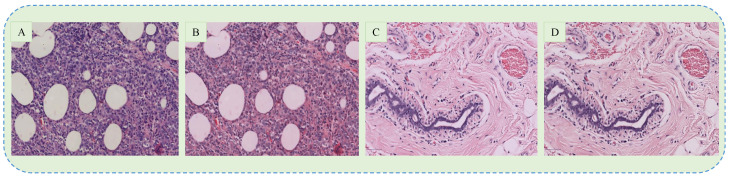
The examples of original (**A**,**C**) and normalized (**B**,**D**) images of carcinoma and non-carcinoma cases.

The dataset used in this paper contains histopathology images of breast cancer stained with H & E, which is widely used to assist pathologists during the microscopic assessment of tissue slides. However, it is difficult to maintain the same staining concentration through all the slides, which results in color differences among the acquired images. These contrast differences may adversely affect the training process of the CNN model and thus the color normalization is usually applied. In this paper, we followed the recent studies [30,31] and employed the approach proposed by [33] for colour normalization. In this method, images are first converted into optical density (OD) by using a logarithmic transformation. Next, singular value decomposition (SVD) is applied to OD tuples to obtain two-dimensional projections with higher variance. Then, the resulting color space transform is applied to the original images. Finally, the histogram of images is stretched in order to cover the lower 90% of data. However, the classification performance of our proposed model deteriorated upon using the normalized images, which is also comprehensively explained in [34]. Eventually, we omitted the stain normalization process and thus used the original images in this paper. The example of original and normalized carcinoma images are shown in Figure 2.

### 3.3. Training Criteria

For the individual and ensemble models, we selected 80% of images for training and the remaining 20% for testing purposes with the same percentage of carcinoma and non-carcinoma images. In this way, 675 images were used for training whereas the remaining 170 images were kept for testing the model. Following [35], we used 5-fold cross-validation on training images which means that 540 images were used for training and 135 images for validation purpose. Again, we have an equal percentage of non-carcinoma and carcinoma images in training and validation. These statistics about training, validation, and testing the models are depicted in Table 2.

### 3.4. Data Augmentation

Image data augmentation is a technique used to expand the dataset by generating modified images during the training process. By employing the *ImageDataGenerator* provided by Keras deep learning library [36], we generate batches of tensor image data with real-time data augmentation. With this type of data augmentation, we want to ensure that our network, when trained, sees new variations of our data at each and every epoch. Firstly, an input batch of images is presented to the *ImageDataGenerator*, which then transforms each image in the batch by a series of random translations, rotations, etc. The rotation which we specified “rotation range = 40” corresponds to a random rotation angle between [−40, 40] degrees. We also set the “width and height shift range = 0.2” which specifies the upper bound of the fraction of the total width by which the image is to be randomly shifted, either towards the left or right for width or up or down for height. Of note, the rotation operation may rotate some pixels out of the image frame and leave behind empty pixels within the frame which must be filled. We used the “reflect mode” in order to fill these empty pixels. Finally, the randomly transformed batch is then returned to the calling function. All these parameters along with their values are shown in Table 3.

### 3.5. VGG Architecture

Pretrained models usually help in a better initialization and convergence when the dataset is comparably small as compared to natural image datasets, and this result has been extensively used in other areas of medical imaging too [31]. To this end, we employed deep CNN-based pretrained model proposed by Visual Geometry Group (VGG) of Oxford University [20]. The VGG model is in fact one of the most influential contributions since it reinforced the notion that CNNs have to have a deep network of layers in order for this hierarchical representation of visual data to work. Although numerous follow-up works made improvements in VGG architecture; however, we used its early layouts in this paper, called VGG16 and VGG19 architectures. These names are given because of the fact that VGG16 contains sixteen weight layers whereas VGG19 carries nineteen weight layers in their basic structures [20].

**Figure 3:**
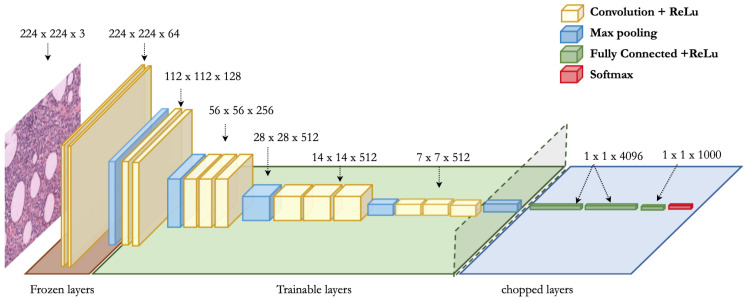
Representation of fine-tuned VGG16 architecture [20]. In fine-tuned VGG16 and VGG19 models, the first block (comprising two convolutional layers and one max-pooling layer) is frozen whereas the rest of layers are trainable. However, in fully-trained VGG16 and VGG19 models, all the five blocks are trainable.

The complete framework of the VGG16 model is portrayed in Figure 3. It is composed of five convolutional blocks and every block has multiple convolution layers (with relu activation), together with a max-pooling layer. It strictly uses 3 × 3 filters with stride and pad of 1, along with 2 × 2 maxpooling layers with stride 2. The basic architecture of VGG19 is the same as that of VGG16, except three extra convolutional layers. We tried four different approaches by using these two pretrained architectures. For fully-trained VGG16, we employed all the five blocks and replaced the last three layers by a single dense layer with 256 nodes, as shown in Figure 3. The final output layer is composed of binary cross-entropy loss function which is mathematically shown in Equation (1). Also, for fine-tuned VGG16, we froze the first block (with two convolutional layers and one max-pooling layer) and used the remaining four blocks for the training purpose. Again, we used one dense layer of 256 nodes along with the same loss function of binary cross-entropy. Similarly, for fully-trained VGG19, we trained all the blocks along with one dense layer of 128 nodes. Also, we froze the first block and trained the remaining blocks in the fine-tuned VGG19 model along with a single dense layer of 128 nodes. The final layer in case of VGG19 is also composed of binary cross-entropy loss function, as shown in Equation (1).

(1) $$Binary\;cross\;entropy=-\frac{1}{m}\sum_{i}^{m}\left(y_{i}*log\left(p(y_{i})\right)+\left(1-y_{i}\right)*log\left(1-p\left(y_{i}\right)\right)\right)$$

### 3.6. Proposed Ensemble Approach

**Figure 4:**
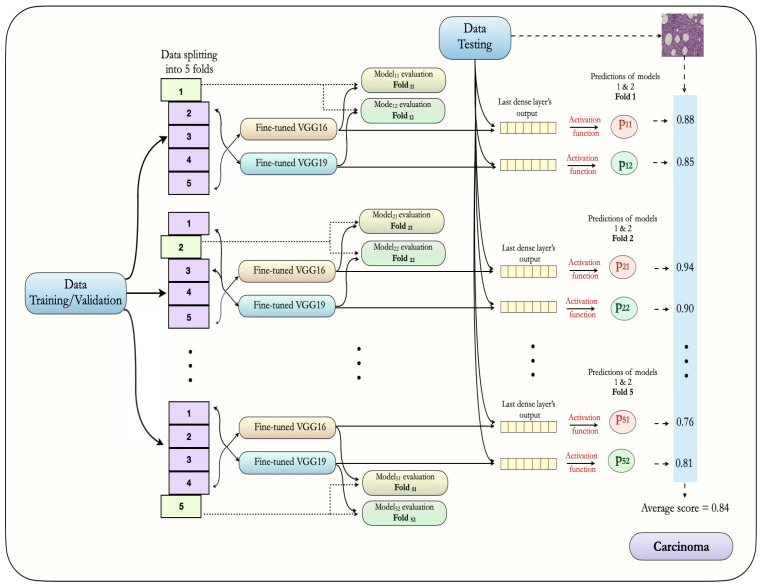
The proposed ensemble architecture using the fine-tuned VGG16 and VGG19 models along with 5-fold cross-validation approach.

The architecture of our proposed ensemble approach is illustrated in Figure 4. It is composed of an ensemble of fine-tuned VGG16 and fine-tuned VGG19 models. First, for both models, training images (80%) are arranged in 5-folds, out of which four are used for training and one is used for model validation or evaluation. Of note, these folds are mutually exclusive and have equal percentages of non-carcinoma and carcinoma images. Also, we used image augmentation during the training process, as described in Table 3. In every fold, we trained each model for 200 epochs; however, we saved weights of the best model only, based on a minimum value of loss function. In this way, we saved the weight for 5 folds for both models. Then, the test images (20%) are utilized in order to make the final prediction in the form of probabilities. The average probability for every class (non-carcinoma and carcinoma) is derived by taking the mean of ten probability values, obtained from 5-fold VGG16 and 5-fold VGG19 models (10 folds in total). In this way, we considered the average probability of both the models in order to classify images into non-carcinoma or carcinoma classes. The final results of our proposed ensemble deep learning approach are discussed in Section 5.

## 4. Experimental Setup

In this section, we explained the experimental environment, followed by the interpretation of evaluation metrics in our proposed model, and finally, we elucidated the tuning of hyperparameters.

### 4.1. Implementation

We implemented all the experiments related to this article by using *Python 3.7.6* along with *TensorFlow 2.1.0* and *Keras 2.2.4* installed on a standard PC with dual Nvidia GeForce GTX 2070 graphical processing unit (GPU) support. Moreover, this PC has a RAM capacity of 32.0 GB and holds a 3.60 GHz Intel^®^ ${Core\;}^{TM}$ i9-9900K processor with 16 logical threads as well as 16 MB of cache memory.

### 4.2. Evaluation Metrics

The overall performance of our proposed model relies on elements of confusion matrix, also called error matrix or contingency table. This evaluation matrix contains four terms, namely, True Positive (TP), False Positive (FP), False Negative (FN), and True Negative (TN). In our problem, TP refers to those images that were correctly classified as carcinoma and the FP represents the non-carcinoma images mistakenly classified as carcinoma. Whereas, the FN represents the images belonging to carcinoma class that were classified as non-carcinoma, and the TN refers to the non-carcinoma images correctly classified. The classification performance of our proposed model was evaluated on the testing set using four performance measures based on confusion matrix, namely, precision, sensitivity (recall), overall accuracy, and F1-score, using python scikit-learn module. These performance measures can be calculated as follow:

- **Precision**: It quantifies exactness of a model, and represents the ratio of carcinoma images accurately classified out of the union of predicted same-class images.
  (2) $$Precision=\frac{TP}{TP+FP}$$
- **Sensitivity**: Sensitivity, also called “recall” computes completeness of a model. It represents the ratio of images accurately classified as carcinoma out of the total number of carcinoma images.
  (3) $$Sensitivity=\frac{TP}{TP+FN}$$
- **Accuracy**: It evaluates correctness of a model, and is the ratio of the number of images accurately classified out of the total number of testing images.
  (4) $$OverallAccuracy=\frac{TP+FN}{TP+TN+FP+FN}$$
- **F1-score**: It represents the harmonic average of precision and recall, and is usually used for the optimization of a model towards either precision or recall.
  (5) $$F1-score=\frac{2*Precision*Recall}{Precision+Recall}$$

### 4.3. Hyperparameter Tuning

Neural networks have a powerful property of learning sophisticated connections between their inputs and outputs automatically [37]. However, some of these connections might be the result of sampling noise, so they can prevail during the training process but could not exist within the real test dataset. This issue may lead to overfitting problems and thus may degrade the prediction performance of a deep learning model [37]. For this very reason, we followed the tuning process of hyperparameters in order to get the generalized performance of our proposed model. The methodology used for the selection of optimal hyperparameters is as follows: First, we selected binary cross-entropy as a loss function for our binary classification problem. Then, Adam (adaptive moment estimation) algorithm [38,39] was used during the training process in order to perform optimization through 200 epochs. At this stage, we tried three different learning rates (0.001, 0.0001, and 0.00001) and three different batch sizes (16, 32, and 64) while keeping in mind the values used in the recently published study [39,40]. During the model training, our primary aim was to minimize the generalization gap between training loss and validation loss, and found that the batch size of 32 worked well together with the learning rate of 0.0001. Also, we used a dropout of 0.3 in order to prevent the model from overfitting during the training process [41]. Next, we saved the weights of five best models based on their minimal validation loss by using a 5-fold cross validation approach. Finally, we employed these weights for the class prediction on the test dataset. Of note, we used the convolutional filters, pooling filters, strides, and padding with their default values mentioned in the original VGG16 and VGG19 architectures [20]. All the optimal values of hyperparameters used in this study are provided in Table 4.

## 5. Results and Discussion

In this section, we evaluated the performances of our proposed deep learning models by taking into consideration the average predicted probabilities. First, we highlighted the performance metrics of individual models and then we discussed the competitiveness of our proposed models with recently published studies, especially in terms of carcinoma classification.

### 5.1. Results of VGG16 Architecture

**Figure 5:**
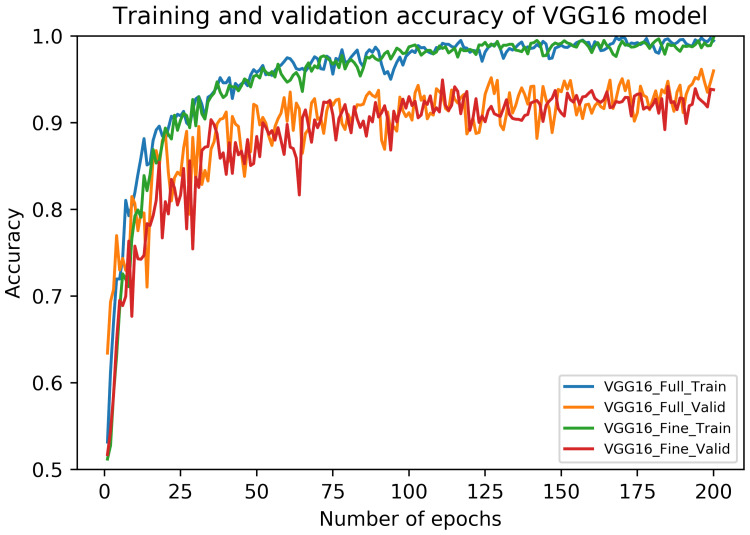
The training and validation accuracy curves of fully-trained and fine-tuned VGG16 models.

**Figure 6:**
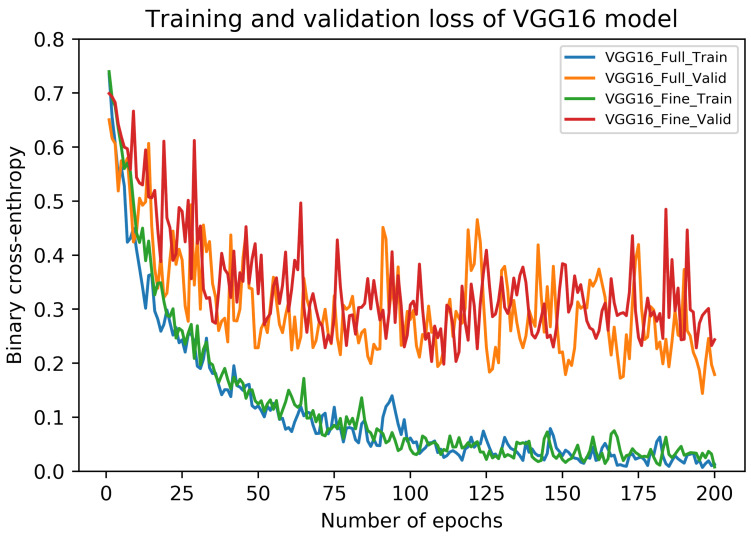
The training and validation loss curves of fully-trained and fine-tuned VGG16 models.

The performance metrics of fully-trained VGG16 architecture on our dataset are shown in Table 5. It can be noticed that these metrics vary across different folds although using the same test samples. Interestingly, the average recall value (sensitivity) of carcinoma class is noted as 94.55% (±2.59). Also, the highest accuracy and F1 score are noted during Fold 1, in contrast to their lowest values during Fold 2. The overall accuracy of the fully-trained VGG16 model is 91.41 (±3.40) along with the average F1 score of 91.38 (±3.42). The accuracy curves of this model are depicted in Figure 5, whereas its loss curves are displayed in Figure 6.

Similar to fully-trained VGG16 architecture, the performance metrics of fine-tuned VGG16 framework are also presented in Table 5. Again, we used the same test set across all the folds. In this case, the average recall value of carcinoma class can be noticed as 94.09% (±3.35). Moreover, the highest accuracy and F1 score are found during Fold 5, whereas their respective lowest values can be seen during Fold 1. Overall, the fine-tuned VGG16 models provided an average accuracy of 91.67% (±3.69) as well as an average F1 score of 91.63% (±3.69). The accuracy curves of this model are also illustrated in Figure 5, whereas its loss curves are presented in Figure 6. Lastly, the training and prediction times of fully-trained and fine-tuned VGG16 models are provided in Table 6.

### 5.2. Results of VGG19 Architecture

**Figure 7:**
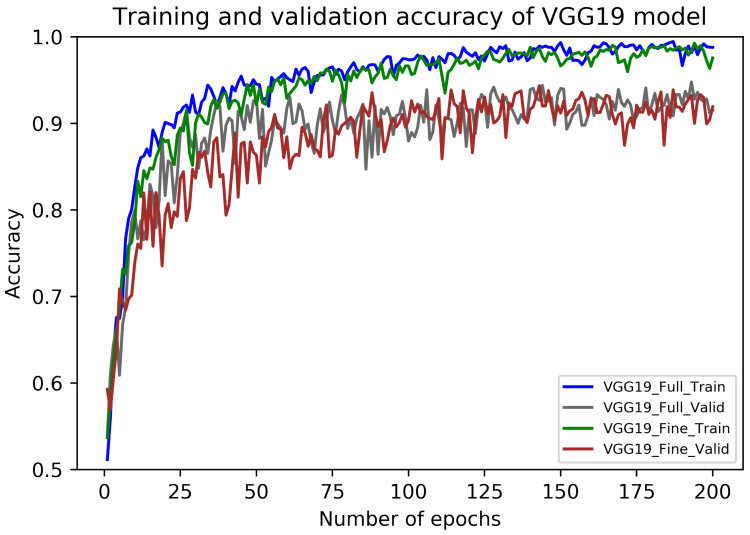
The training and validation accuracy curves of fully-trained and fine-tuned VGG19 models.

**Figure 8:**
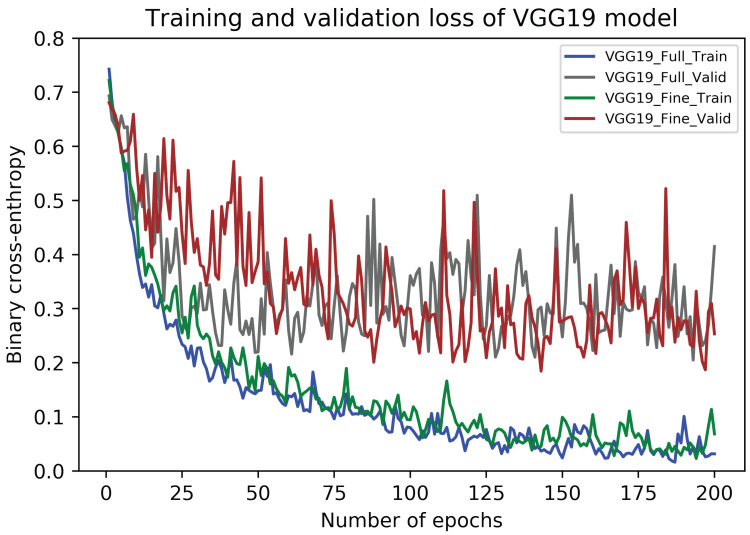
The training and validation loss curves of fully-trained and fine-tuned VGG19 models.

The performance metrics of fully-trained VGG19 architecture on our dataset are presented in Table 7. In this case, the average recall value (sensitivity) for carcinoma images is 95.45% (±3.41) which is 0.9 percentage points higher than that of the fully-trained VGG16 model. Also, the maximum values of the accuracy and an F1 scores occurred during Fold 3, whereas their minimum values found during Fold 4. Finally, the overall accuracy of the fully-trained VGG19 model is 90.35% (±1.35) in together with the average F1 score of 90.31% (±1.35). The accuracy curves of this model are illustrated in Figure 7, whereas its loss curves are portrayed in Figure 8.

Similar to the fully-trained VGG19 model, the performance metrics of fine-tuned VGG19 architecture are portrayed in Table 7. The average recall value for carcinoma cases is 95.68% (±3.15) which reflects 1.59 percentage points higher than that of the fine-tuned VGG16 model. In this case, the highest values of accuracy and an F1 score are noted for Fold 3 and 4, whereas their low values occurred during Fold 1. The average accuracy and F1 score in this case are 91.67% (±2.99) and 91.63% (±3.03), respectively. The accuracy curves of this model are also presented in Figure 7, whereas its loss curves are shown in Figure 8. Finally, like the VGG16 models, the training and prediction times of fully-trained and fine-tuned VGG19 frameworks are also given in Table 6.

### 5.3. Results of Ensemble VGG16 and VGG19

The performance metrics of the ensemble VGG16 and VGG19 framework are shown in Table 8. In this approach, we ensemble the fully-trained VGG16 and VGG19 architectures and the fine-tuned VGG16 and VGG19 frameworks by taking the average of output probabilities among all the folds in the aforementioned architectures. Interestingly, the recall value for the carcinoma class is noted as the same (97.73%) in both fully-trained and fine-tuned ensemble approaches. However, the fine-tuned approach offered high accuracy and F1 score (overall) compared to the fully-trained approach, as shown in Table 8.

### 5.4. Discussion

The effectiveness of our proposed ensembling approach can be compared with various state-of-the-art studies used for the classification of breast cancer histopathology images. Most of these novel deep learning approaches are based on BreakHis dataset [27]. For instance, Spanhol et al. [28] employed a variant of AlexNet [19] for the classification of benign and malignant images of BreakHis dataset [27]. The authors used sum, product and maximum fusions rules along with different patch sizes and reported an image level accuracy of 84.0% (±3.2) for 200× image magnification. In the following year, Bayramoglu et al. [29] proposed a magnification independent approach for BreakHis dataset. Specifically, the authors presented “single task CNN” and “multi-task CNN” frameworks, where the former predicts malignancy and the latter predicts malignancy as well as the magnification level in the benign and malignant images. For 200× magnification, the authors reported an accuracy of 84.63% (±2.72) and 82.56% (±3.49) for single task CNN and multi-task CNN, respectively. Both of these studies [28,29] reported better classification performance than the traditional hand-crafted machine learning approaches. In comparison with Spanhol et al. [28] and Bayramoglu et al. [29], our approach shows better classification performance despite using a comparatively small dataset. Recently, Han et al. [42] proposed a structured deep learning model called class structure-based deep CNN (CSDCNN) for the classification of benign and malignant histopathology images of breast cancer, and reported an accuracy of 96.7% (±2.0) on BreakHis dataset for 200× magnification factor. Similarly, Nahid et al. [43] first used clustering algorithm in order to retrieve the statistical and geometrical clusters hidden in the histopathology images. The authors then evaluated the effect of deep CNN in together with short-term memory (LSTM) network for the efficient classification of benign and malignant images, and thus achieved an accuracy of 91.0% on BreakHis dataset for 200× magnification. Lastly, Daniz et al. [44] employed fine-tuned AlexNet [19] and VGG16 [20] models for the classification of breast cancer histopathology images. The authors followed 5-fold cross-validation approach and reported a maximum accuracy of 91.37% (±1.72) when using fine-tuned AlexNet [19] on BreakHis dataset for 200× magnification. These state-of-the-art studies [28,29,42,43,44] along with other novel frameworks are comprehensively reviewed in [45]. Although having a small dataset, our results are still competitive with the novel deep learning frameworks [28,29,42,43,44,45]. In summary, the results demonstrated that our proposed ensemble deep learning model can retrieve various multi-level and multi-scale features from histopathology images of breast cancer. It also became clear from the comparison process that the results of our proposed architecture is competitive with numerous state-of-the-art studies using comparably bigger datasets.

## 6. Conclusions

In this paper, we presented an ensemble deep learning approach for the classification of breast cancer histopathology images using our collected dataset. The main objective of this work was to effectively classify carcinoma images. We found that it could be better to use the average predicted probabilities of two individual models. To this end, we employed an ensemble of fine-tuned VGG16 and VGG19 models and achieved a relatively more robust model. The proposed ensemble approach provides competitive performance on the classification of complex-natured histopathology images of breast cancer. However, our collected dataset is comparatively small in contrast to the datasets used in numerous state-of-the-art studies. Also, our dataset contains merely two-class images. The future indications of this study include the extension of our dataset and the inclusion of images for multi-class classification problems. Also, other pretrained models need to be included in the future work. Finally, it will be interesting to apply similar ensemble criteria to histopathology images of different cancers, such as lung cancer.

## Figures and Tables

**Table 1:** Characteristics of our proposed dataset.

Images	Quantity	Color Model	Staining
carcinoma	437	RGB	H & E
non-carcinoma	408	RGB	H & E
Total	845	RGB	H & E

**Table 2:** Criteria for the selection of training, validation, and test images.

	No. of Images	Percentage
Training	540	64%
Validation	135	16%
Test	170	20%
Total	845	100%

**Table 3:** Parameters of data augmentation.

Parameters of Image Augmentation	Values
Zoom range	0.2
Rotation range	40
Width shift range	0.2
Height shift range	0.2
Horizontal flip	True
Fill mode	Reflect

**Table 4:** Hyperparameters used in the individual and an ensemble models.

Hyperparameters	VGG16 with Data Augmentation	VGG19 with Data Augmentation
Train approach	5-fold cross-validation	5-fold cross-validation
Optimizer	Adam	Adam
Loss function	Binary cross-entropy	Binary cross-entropy
Learning rate	0.0001	0.0001
Batch size	32	32
Convolution	3×3 with stride 1	3×3 with stride 1
Padding	Same	Same
Pooling	2×2 max-pooling with stride 2	2×2 max-pooling with stride 2
Epochs	200	200
Drop out	0.3	0.3
Regularizer	N/A	N/A
Architecture	Fully-trained and Fine-tuned	Fully-trained and Fine-tuned

**Table 5:** Performance metrics of VGG16 architecture on our dataset.

Architecture	Folds	Confusion Matrices			Performance Evaluation (%)				Average (%)	
		Predict → Actual ↓	NC	C	Precision	Recall	F1	Test	Acc.	F1
**Fully-Trained** **VGG16**	Fold 1	non-carcinoma	75	7	97.40	91.46	94.34	82	**94.71**	**94.70**
		carcinoma	2	86	92.47	97.73	95.03	88		
	Fold 2	non-carcinoma	65	17	90.28	79.27	84.42	82	**85.88**	**85.80**
		carcinoma	7	81	82.65	92.05	87.10	88			
	Fold 3	non-carcinoma	73	9	93.59	89.02	91.25	82	**91.76**	**91.75**
		carcinoma	5	83	90.22	94.32	92.22	88			
	Fold 4	non-carcinoma	70	12	95.89	85.37	90.32	82	**91.18**	**91.13**
		carcinoma	3	85	87.63	96.59	91.89	88			
	Fold 5	non-carcinoma	78	4	91.76	95.12	93.41	82	**93.53**	**93.53**
		carcinoma	7	81	95.29	92.05	93.64	88			
	Avg.	non-carcinoma	–	–	93.78	88.05	90.75	82	**91.41**	**91.38**
		carcinoma	–	–	89.65	94.55	91.98	88			
**Fine-Tuned** **VGG16**	Fold 1	non-carcinoma	67	15	87.01	81.71	84.28	82	**85.29**	**85.27**
		carcinoma	10	78	83.87	88.64	86.19	88		
	Fold 2	non-carcinoma	74	8	92.50	90.24	91.36	82	**91.76**	**91.76**
		carcinoma	6	82	91.11	93.18	92.13	88		
	Fold 3	non-carcinoma	76	6	95.00	92.68	93.83	82	**94.12**	**94.11**
		carcinoma	4	84	93.33	95.45	94.38	88		
	Fold 4	non-carcinoma	73	9	96.05	89.02	92.41	82	**92.94**	**92.92**
		carcinoma	3	85	90.43	96.59	93.41	88		
	Fold 5	non-carcinoma	75	7	96.15	91.46	93.75	82	**94.12**	**94.11**
		carcinoma	3	85	92.39	96.59	94.44	88		
	Avg.	non-carcinoma	–	–	93.34	89.02	91.13	82	**91.67**	**91.63**
		carcinoma	–	–	90.23	94.09	92.11	88		

**Table 6:** The training and prediction times of fully-trained and fine-tuned models.

Model	Single Training Time	5-Fold Training Time	Prediction Time
Fully-trained VGG16	17 min 50 s	89 min	30 s
Fine-tuned VGG16	17 min 25 s	87 min	31 s
Fully-trained VGG19	20 min 40 s	103 min	35 s
Fine-tuned VGG19	19 min 55 s	99 min	36 s

**Table 7:** Performance metrics of VGG19 architecture on our dataset.

Architecture	Folds	Confusion Matrices			Performance Evaluation (%)				Average (%)	
		Predict → Actual ↓	NC	C	Precision	Recall	F1	Test	Acc.	F1
**Fully-Trained** **VGG19**	Fold 1	non-carcinoma	66	16	98.51	80.49	88.59	82	**90.00**	**89.89**
		carcinoma	1	87	84.47	98.86	91.10	88		
	Fold 2	non-carcinoma	71	11	94.67	86.59	90.45	82	**91.18**	**91.15**
		carcinoma	4	84	88.42	95.45	91.80	88		
	Fold 3	non-carcinoma	69	13	98.57	84.15	90.79	82	**91.76**	**91.70**
		carcinoma	1	87	87.00	98.86	92.55	88		
	Fold 4	non-carcinoma	69	13	90.79	84.15	87.34	82	**88.24**	**88.21**
		carcinoma	7	81	86.17	92.05	89.01	88		
	Fold 5	non-carcinoma	73	9	91.25	89.02	90.12	82	**90.59**	**90.58**
		carcinoma	7	81	90.00	92.05	91.01	88		
	Avg.	non-carcinoma	–	–	94.76	84.88	89.46	82	**90.35**	**90.31**
		carcinoma	–	–	87.21	95.45	91.09	88		
**Fine-Tuned** **VGG19**	Fold 1	non-carcinoma	64	18	98.46	78.05	87.07	82	**88.82**	**88.67**
		carcinoma	1	87	82.86	98.86	90.16	88		
	Fold 2	non-carcinoma	75	7	93.75	91.46	92.59	82	**92.94**	**92.94**
		carcinoma	5	83	92.22	94.32	93.26	88		
	Fold 3	non-carcinoma	75	7	97.40	91.46	94.34	82	**94.71**	**94.70**
		carcinoma	2	86	92.47	97.73	95.03	88		
	Fold 4	non-carcinoma	76	6	96.20	92.68	94.41	82	**94.71**	**94.70**
		carcinoma	3	85	93.41	96.59	94.97	88		
	Fold 5	non-carcinoma	71	11	89.87	86.59	88.20	82	**88.82**	**88.81**
		carcinoma	8	80	87.91	90.91	89.39	88		
	Avg.	non-carcinoma	–	–	95.14	88.05	91.32	82	**92.00**	**91.96**
		carcinoma	–	–	89.77	95.68	92.56	88		

**Table 8:** Performance metrics of ensemble VGG16 and VGG19 architectures.

Ensemble Method	Confusion Matrices			Performance Evaluation (%)				Average (%)	
	Predict → Actual ↓	NC	C	Precision	Recall	F1	Test	Accuracy	F1
**Full-Trained**	non-carcinoma	73	9	97.33	89.02	92.99	82	**93.53**	**93.51**
**VGG16+VGG19**	carcinoma	2	86	90.53	97.73	93.99	88			
**Fine-Tuned**	non-carcinoma	76	6	97.44	92.68	95.00	82	**95.29**	**95.29**
**VGG16+VGG19**	carcinoma	2	86	93.48	97.73	95.56	88			

## Abbreviations

The following abbreviations are used in this manuscript:

GBD	Global Burden of Disease
MRI	Magnetic Resonance Imaging
CNN	Convolutional Neural Network
LSTM	Long Short Term Memory
KNN	K-Nearest Neighbor
NB	Naive Bayes
DT	Decision Tree
SVM	Support Vector Machine
H&E	Hematoxylin and Eosin
SVD	Singular Value Decomposition
OD	Optical Density
WSI	Whole Slides Image
ER	Estrogen Receptor
PR	Progesterone Receptor
HER2	Human Epidermal growth factor Receptor-2
